# Bile Acid Dysregulation in Parkinson’s Disease: Longitudinal Changes and Altered Metabolic Interactions

**DOI:** 10.3390/biom16060875

**Published:** 2026-06-15

**Authors:** Andrea Ižarik Verešpejová, Marián Grendár, Martin Kertys, Natália Huňarová, Li Sheng Chien, Milan Grofik, Michaela Škorvanová, Jakub Šofranko, Nela Žideková, Egon Kurča, Martin Kolísek

**Affiliations:** 1 Department of Medical Biochemistry, Jessenius Faculty of Medicine in Martin, Comenius University, 036 01 Bratislava, Slovakia; verespejova7@uniba.sk (A.I.V.); hunarova2@uniba.sk (N.H.); 2 Biomedical Centre Martin, Jessenius Faculty of Medicine in Martin, Comenius University, 036 01 Bratislava, Slovakiamichaela.skorvanova@uniba.sk (M.Š.); nela.zidekova@uniba.sk (N.Ž.); 3 Department of Pharmacology, Jessenius Faculty of Medicine in Martin, Comenius University Bratislava, 036 01 Bratislava, Slovakia; martin.kertys@uniba.sk; 4 Department of Molecular Biology, Faculty of Natural Sciences in Bratislava, Comenius University, 841 04 Bratislava, Slovakia; chien1@uniba.sk; 5 Clinic of Neurology, Martin University Hospital, 036 01 Martin, Slovakia; milangrofik@gmail.com (M.G.); egon.kurca@uniba.sk (E.K.)

**Keywords:** Parkinson’s disease, bile acids, gut-brain axis, biomarkers, longitudinal study

## Abstract

Bile acids (BA) are increasingly recognized as signaling molecules involved in metabolic regulation and inflammatory processes, both of which are relevant to Parkinson’s disease (PD). However, their role in PD and disease progression remains unclear. In this study, plasma BA profiles were analyzed in 113 participants, including early- and advanced-stage PD patients and age- and sex-matched controls, across three time points over three years. Targeted metabolomics using LC-MS was applied to quantify 20 BA, complemented by analyses of functional ratios, including unconjugated/conjugated and hydrophobic/hydrophilic BA ratios and correlation patterns between BA species. Although most individual BA did not show consistent longitudinal changes, pooled analysis identified significant differences in the unconjugated/conjugated BA ratio between PD patients and controls. In contrast, the hydrophobic/hydrophilic ratio did not differ significantly between groups. Correlation analysis revealed differences in selected BA interrelationships, particularly involving primary and secondary BA, while the overall network structure remained largely preserved. These results indicate that BA metabolism in PD might be characterized rather by subtle, distributed alterations than pronounced changes in individual metabolites. BA profiling may therefore contribute to a broader metabolic characterization of PD, but its utility as a standalone biomarker appears limited.

## 1. Introduction

Bile acids (BA) are steroid-derived molecules synthesized from cholesterol and represent major functional components of bile [1]. Following conjugation with glycine or taurine, they are secreted into bile and released into the intestine, where they facilitate lipid digestion and absorption [1].

Two major pathways of BA synthesis have been described: the classical (neutral) and the alternative (acidic) pathway [2,3]. The classical pathway, responsible for producing primary BA such as cholic acid (CA) and chenodeoxycholic acid (CDCA), takes place in the liver. In contrast, the alternative pathway can occur in multiple tissues, with its initial steps localized in mitochondria [3]. Following their synthesis, most primary BA are activated by coenzyme A, which facilitates their subsequent conjugation. After secretion into the small intestine, bile acids undergo further modifications by gut microbiota, resulting in the formation of secondary BA such as deoxycholic acid (DCA) and lithocholic acid (LCA) [4,5,6].

In recent years, it has become increasingly evident that the spectrum of BA in humans is broader than previously assumed. However, muricholic acids have traditionally been regarded as the primary BA characteristic of rodents; advances in analytical techniques have enabled their detection, and they have also been identified in human biological samples [7,8]. Their presence likely reflects complex interactions between the host and the gut microbiota, as well as potential contributions from dietary sources or alternative metabolic pathways [9].

Beyond their role in lipid digestion and absorption, BA are closely linked to the gut microbiome [4,10]. Gut microbiota contributes to BA deconjugation and conversion of primary BA into secondary BA, thereby influencing BA composition, concentration, and pool size [5,6,11,12]. In turn, BA can modulate microbial growth and composition, forming a bidirectional gut–microbiome–BA axis relevant to host metabolism- and disease-related processes [11,12]. BA also function as hormone-like signaling molecules [3]. Dysregulation of BA metabolism has been implicated as a potential risk factor in neurodegenerative diseases, including PD and Alzheimer’s disease [13,14].

PD is a progressive neurodegenerative disorder characterized by both motor and non-motor symptoms, with pathological accumulation of α-synuclein in the central nervous system [15,16]. Increasing evidence indicates that gastrointestinal α-synuclein may impair intestinal barrier function and contribute to disease progression via the gut–brain axis, as demonstrated by experimental studies showing that enteric α-synuclein induces inflammatory responses and disrupts intestinal epithelial integrity at early, potentially prodromal stages of the disease [14,17,18].

Alterations in gut microbiota composition in PD patients have been associated with the dysregulation of BA metabolism, leading to shifts in the balance between pro-inflammatory and protective BA species [19,20,21]. Such dysregulated BA metabolites may be linked to neuroinflammation, mitochondrial dysfunction, and oxidative stress [22,23]. Moreover, certain secondary BA are capable of crossing the blood–brain barrier and directly modulating neuronal function [24,25]. Chronic constipation, a common non-motor symptom of PD, may further exacerbate these pathological processes by disrupting intestinal barrier integrity and increasing intestinal permeability. Clinical studies have demonstrated that patients with PD exhibit significantly increased gut permeability, which is associated with the presence of bacterial products and α-synuclein in the intestinal mucosa. This facilitates the translocation of toxins and pro-inflammatory molecules into the systemic circulation and subsequently into the central nervous system [26].

Despite increasing interest in the role of BA metabolism in PD, available human studies remain limited, and findings are inconsistent. In particular, it remains unclear whether BA profiles differ between PD patients and healthy controls, whether these differences are related to disease stage, and whether longitudinal changes can be detected over time.

In the present study, we performed longitudinal profiling of plasma BA in patients with PD and age- and sex-matched healthy controls across three time points. We assessed individual BA concentrations, functional ratios, and correlation patterns between BA species to evaluate whether PD is associated with alterations in BA composition or broader BA metabolic relationships.

## 2. Materials and Methods

### 2.1. Study Design and Cohorts

A total of *N* = 113 participants were included in the study and divided into four independent groups: patients with early-stage idiopathic Parkinson’s disease (PD1, *n* = 29), patients with advanced-stage idiopathic Parkinson’s disease (PD2, *n* = 28), and two groups of healthy controls (HC1, *n* = 28; HC2, *n* = 28). The study was conducted over a three-year period. The two healthy control groups were separated to provide age- and sex-matched reference groups for the early-stage PD group (HC1 matched to PD1) and the advanced-stage PD group (HC2 matched to PD2). This design was used to reduce confounding by age and sex when comparing PD subgroups differing in disease stage. HC1 and HC2 were not intended to represent biologically distinct control populations.

Inclusion criteria for PD patients were the diagnosis of idiopathic Parkinson’s disease established by a neurologist according to standard clinical criteria, age ≥ 18 years, ability to provide written informed consent and availability for longitudinal follow-up. Patients were stratified into early- and advanced-stage PD groups based on HY stage and clinical assessment.

Inclusion criteria for healthy controls were the absence of clinically diagnosed neurodegenerative, metabolic, and/or inflammatory disease at the time of recruitment, age- and sex-matching to the PD cohorts as closely as possible and ability to provide written informed consent.

Exclusion criteria for all participants included acute infection or inflammatory disease at the time of sampling, known active malignancy, severe hepatic or biliary disease, severe renal impairment, and inability to provide informed consent.

Demographic characteristics, including age and sex distribution, drugs, and other clinical parameters were summarized for all groups (Table 1). Age is presented as mean ± standard deviation (SD). Differences in age between groups were assessed using one-way analysis of variance (ANOVA), while sex distribution was compared using the chi-square test (*χ*^2^ test). Clinical parameters (Hoehn–Yahr scale (HY); Unified Parkinson’s disease rating scale part 3 (Motor examination) (UPDRS3), levodopa equivalent daily dose (LEDD), Montreal Cognitive Assessment (MoCA), and Parkinson’s disease questionnaire 39 (PDQ39)) were evaluated only in patients with PD and are reported as mean ± SD. Information on antiparkinsonian medication was available for PD patients and included levodopa-containing preparations, dopamine agonists, MAO-B inhibitors, amantadine, and anticholinergic therapy. Moreover, levodopa equivalent daily dose was included among clinical variables in the PD cohort. A *p*-value < 0.05 was considered statistically significant.

### 2.2. Sample Collection and Plasma Isolation

Fresh peripheral venous blood samples were obtained from the Neurology Clinic of the University Hospital in Martin. All participants underwent blood collection in the fasting state into EDTA-containing tubes (BD Vacutainer, New Jersey, USA). Plasma was isolated from the whole 3 mL of blood by centrifugation at 2000× *g* for 10 min at 4 °C, followed by a second centrifugation under the same conditions to obtain cell-free plasma. Plasma samples were collected under fasting conditions to reduce variability, as both circadian rhythms and postprandial metabolic changes can influence the levels of signaling molecules.

### 2.3. Quantitative Analysis of 20 Bile Acids via LC-MS-Targeted Metabolomics

BA were quantified using the Bile Acids kit (Biocrates Life Science, Innsbruck, Austria) according to the manufacturer’s instructions. The kit was processed using an LC-MS system consisting of an ACQUITY UPLC™ I-Class liquid chromatography system (Waters, Prague, Czech Republic), comprising a flow-through-needle sample manager, a binary solvent manager pump and column manager, coupled with XEVO TQ-S triple quadrupole mass spectrometer (Waters, Prague, Czech Republic). Chromatographic and mass spectrometry conditions were described in detail in the provided user manual (covering aspects such as mobile phase composition, MRM transitions, etc.).

Briefly, 10 μL of the supplied internal standard solution was pipetted into each well on the filter spot of the 96-well extraction plate and dried under a gentle stream of nitrogen for 5 min. Subsequently, 10 μL of plasma samples, quality control (QC) samples, and calibration standards were pipetted, followed by another nitrogen drying step. BA were extracted using 100 μL of methanol by shaking for 20 min at 600 rpm. Next, the plate was centrifuged for 2 min at 500× *g* to elute the methanol extracts into the capture plate, and 60 μL of water was added to each well. The diluted extracts were chromatographically separated on a proprietary reversed-phase UHPLC column supplied by the kit manufacturer (Biocrates Life Sciences, Innsbruck, Austria) and detected in multiple reaction monitoring mode by a mass spectrometer. The mass spectrometer signals were obtained using MassLynx software version 4.2 (Waters, Prague, Czech Republic). The data from MassLynx were then analyzed and quantified with MetIDQ software version Oxygen-DB 110-3005-290 (Biocrates Life Sciences AG, Innsbruck, Austria), which is included with the kit. Finally, intra-plate correction was performed by normalizing medium QC samples using the QC2-level sample (analyzed in five technical replicates).

### 2.4. Unconjugated/Conjugated Bile Acid Ratio (U/C Ratio)

The ratio of unconjugated to conjugated BA (U/C ratio) was calculated for each participant by summing the concentrations of individual BA within each category and computing their ratio.

The classification of individual BA into unconjugated and conjugated categories is shown in Table 2. The U/C ratio was calculated as follows: Core U/C ratio: log_2_ (Σ unconjugated BA/Σ conjugated BA).

Extended U/C ratio: log_2_ (Σ unconjugated BA, including HDCA, MCAα, MCAβ, and MCAω/Σ conjugated BA, including TMCAα+β).

### 2.5. Hydrophobic/Hydrophilic Bile Acid Ratio (HFO/HFI Ratio)

BA were categorized into hydrophobic and hydrophilic species based on their physicochemical properties. The core ratio included major human BA, where UDCA- and CA-derived species were classified as hydrophilic, while CDCA-, DCA-, and LCA-derived species were classified as hydrophobic. An extended ratio was additionally calculated by including muricholic BA (MCAα, MCAβ, and their conjugates), tauromuricholic acids (TMCA), and hyodeoxycholic acid (HDCA) among hydrophilic BA. Muricholic BA were classified as hydrophilic due to their lower membrane toxicity and their role in reducing the overall hydrophobicity of the bile acid pool, as demonstrated in experimental models [27,28].

The classification of BA into hydrophobic and hydrophilic categories is shown in Table 3. The HFO/HFI ratio was calculated as follows: core HFO/HFI ratio: log_2_ (Σ hydrophobic BA/Σ hydrophilic BA).

Extended HFO/HFI ratio: log_2_ (Σ hydrophobic BA/Σ hydrophilic BA, including MCAα, MCAβ, MCAω, TMCAα+β, and HDCA).

### 2.6. Statistical Analysis of BA Ratios

BA ratios were log_2_-transformed to reduce skewness and to enhance interpretability in terms of fold changes.

Distributional properties of the data were assessed using graphical methods (Q-Q plots) supported by the Shapiro–Wilk test. Although log_2_ transformation improved distributional symmetry, visual inspection of Q-Q plots revealed residual deviations from normality, particularly in PD groups. Therefore, non-parametric statistical tests were applied throughout.

Differences between the four study groups (PD1, PD2, HC1, HC2) at individual time points (T0–T2) were evaluated using the Kruskal–Wallis test. When appropriate, pairwise comparisons were performed using Dunn’s post hoc test with Bonferroni correction to account for multiple testing.

For pooled comparisons between PD patients (PD1 + PD2) and controls (HC1 + HC2), the Mann–Whitney U test was used. Statistical analyses were performed using Jamovi software (version 2.6; https://www.jamovi.org).

### 2.7. Statistics Analysis

Data exploration and analysis were performed in R (RRID:SCR_001905), version 4.4.0, using the lcmm package [29,30]. Continuous variables were summarized as medians with interquartile ranges. Categorical variables were reported as counts and percentages. Latent class linear mixed models (LCMM; also known as heterogeneous linear mixed models) were fitted to long-format data to evaluate associations between predictors and the response variable.

For the four groups (HC1, HC2, PD1, PD2), we fitted a 1-class model (m1: response ~ Year + Year^2^ + Age + Gender + (1|subject)) and a 2-class model (m2) with identical functional form but allowing two latent classes. Model selection between m1 and m2 was based on log-likelihood and Bayesian Information Criterion (BIC). Class homogeneity was quantified using class membership percentages and entropy. The selected model (m2 in all cases) was used to predict response values, from which estimated marginal means and 95% confidence intervals were computed (in-house implementation), followed by post-hoc Wald tests (in-house implementation. For the two PD groups (PD1, PD2), 1-class and 2-class models followed the form: response ~ Year + Year^2^ + Age + Gender + HYs + UPDRS3s + LEDDs + MoCAs + PDQ39s + (1|subject), where HY, UPDRS3, LEDD, MoCA, and PDQ39 were scaled. These models were analyzed as described for the four-group analysis.

## 3. Results

**Figure 1:**
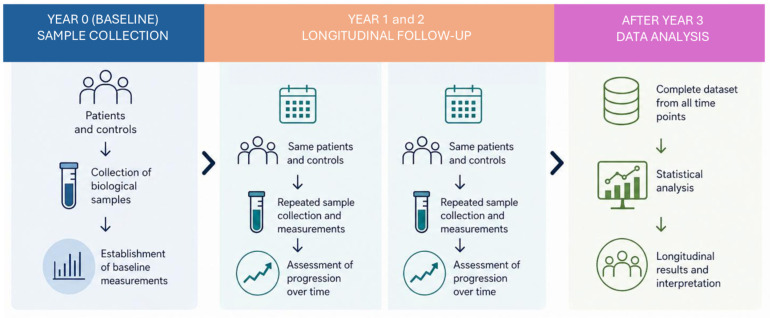
Graphical design of study. Samples were collected in year 0 (T0; baseline) and the same patients and control participants were followed for 2 additional years (T1 and T2)) to assess progression over time. After 3 consecutive years of data collection and measurements were analyzed all data to generate longitudinal results.

This study aimed to identify robust and potentially predictive markers of idiopathic PD through quantitative profiling of 20 BA using a combined cross-sectional and longitudinal design across three time points (years 0 as a baseline (T0), year 1 (T1) and year 2 (T2)), enabling assessment of both group differences and temporal dynamics (Figure 1).

BA profiles were analyzed in relation to clinical parameters, including disease severity, motor and cognitive function, and treatment variables. To reduce clinical heterogeneity, cohorts were stratified a priori into subgroups (PD1, PD2; HC1, HC2) based on HY stage, allowing comparison between earlier and more advanced stages of PD.

Longitudinal analysis revealed substantial intra-individual variability. The applied modelling framework therefore identified dominant subsets with consistent temporal profiles and smaller subsets with heterogeneous patterns, which were analyzed separately. The results presented here focus on the dominant subsets, representing the most robust and biologically interpretable BA dynamics.

### 3.1. Prospective Changes in Concentrations of BA over Time

**Figure 2:**
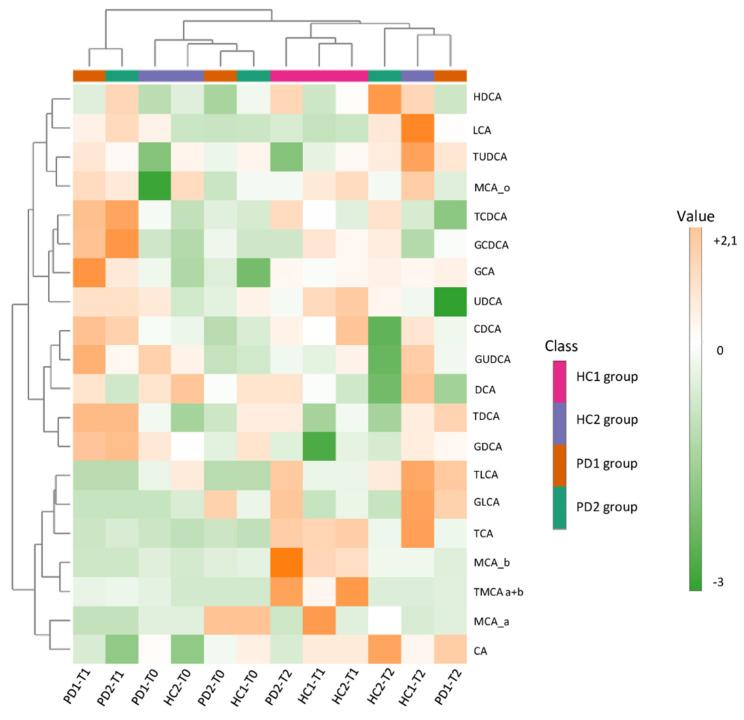
Clustered heatmap of mean bile acid concentrations across time points (T0–T2) in patient (PD1, PD2) and control (HC1, HC2) groups. Data were processed using MetaboAnalyst 6.0 and auto-scaled (mean-centered and variance-scaled). Rows represent individual BA (*n* = 20), and columns represent groups at each time point. The color scale indicates relative concentration levels, where white represents 0, green indicates lower (negative) values, and orange indicates higher (positive) values. Hierarchical clustering was performed using Euclidean distance and average linkage.

Mean concentrations of 20 BA (µmol/L) were measured over three consecutive years in two patient groups (PD1, PD2) and two control groups (HC1, HC2). Several BA exhibited minor to pronounced temporal changes. Temporal changes in selected BA are summarized in Table 4, while the overall dynamics are visualized in the heatmap (Figure 2).

Among individual BA, the most prominent time-dependent changes were observed for LCA across all four groups (PD1, ∆T0–T1 *p* = <0.001; PD2, ∆T0–T1 *p* = <0.001), as well as for HDCA (PD1, ∆T0–T1 *p* = 0.019; PD2, ∆T0–T1 *p* = <0.001) and muricholic acids. In contrast, the remaining BA were largely stable over time or showed only minor, non-significant changes.

Primary BA showed a decreasing trend in the control groups, particularly in HC1 between T0 and T2 (CA, HC1 ∆T0–T2 = −0.08 µmol/L; CDCA, HC1 ∆T0–T2 = −0.16 µmol/L), while only minimal changes were observed in HC overall. In contrast, concentrations of these BA increased in patient groups over the same interval (CA, PD1 ∆T0–T2 = 0.10 µmol/L; CDCA, PD1 ∆T0–T2 = 0.02 µmol/L; CA, PD2 ∆T0–T2 = 0.13 µmol/L; CDCA, PD2 ∆T0–T2 = 0.14 µmol/L).

When categorized based on physicochemical properties, hydrophobic BA showed greater variability over time and between groups. In contrast, hydrophilic BA were more stable, exhibited an increasing trend between T0 and T2, and showed minimal intergroup differences.

The highest heterogeneity was observed among conjugated BA, particularly TUDCA and TCDCA; however, these changes were small and did not reach statistical significance (Table 4).

For an overall visualization of temporal changes across all BA in all four groups, a clustered heatmap was generated using MetaboAnalyst 6.0. The heatmap illustrates relative changes in mean BA concentrations across time points (T0–T2) within each group, because the primary aim was to capture group-level temporal trends more than inter-individual variability. Data were auto-scaled (mean-centered and divided by the standard deviation) prior to analysis to enable comparison across metabolites. Rows represent 20 bile acids, and columns correspond to individual groups at each time point of study.

Hierarchical clustering revealed three main BA clusters with distinct temporal patterns (Figure 2), with time-dependent changes more representing the dominant source of variation than clear separation between PD and control groups.

The upper cluster, comprising HDCA, LCA, and TUDCA, showed relatively higher levels at earlier time points, followed by stabilization or a mild decrease over time across both PD and control groups. This pattern indicates an early shift in BA composition that plateaus longitudinally, not progressively diverging between groups.

A second cluster, enriched in conjugated BA such as TCDCA, GCDCA, and GCA, exhibited heterogeneous patterns without a consistent temporal trend or clear group-specific separation. This observation is consistent with the absence of statistically significant changes in univariate analyses, indicating the relative stability of these metabolites.

The central cluster was characterized by increased variability across time points and groups. Within this cluster, UDCA showed more pronounced differences between PD1 and HC1 groups across all time points (T0 *p* = 0.007; T1 *p* = <0.001; T2 *p* = 0.025), whereas no significant differences were observed between HC2 and PD2. This supports a group-dependent effect that is not consistent across cohorts. Other BA within this cluster (e.g., CDCA, DCA) did not exhibit consistent directional trends, indicating heterogeneous regulation.

The lower cluster, including TLCA, GLCA, TCA, muricholic acids, and CA, was characterized by lower levels at earlier time points followed by a gradual increase toward later time points across groups, with a more pronounced shift in PD groups (ΔT0–T2: positive trend). This temporal increase was most evident for TLCA and TCA. TLCA also showed statistically significant differences between PD and control groups in previous analyses (HC1 vs. PD1 at T1: *p* = 0.031; HC2 vs. PD2 at T2: *p* = 0.004), supporting the observed pattern.

Overall, hierarchical clustering did not reveal a clear separation between the PD and control groups. Instead, temporal dynamics were the primary driver of BA variability, with coordinated longitudinal changes observed across multiple BA classes. While certain metabolites exhibited group-specific differences, these effects were not consistent across cohorts and were secondary to the overall time-dependent trends.

### 3.2. Conjugated/Unconjugated Bile Acids Ratio

**Figure 3:**
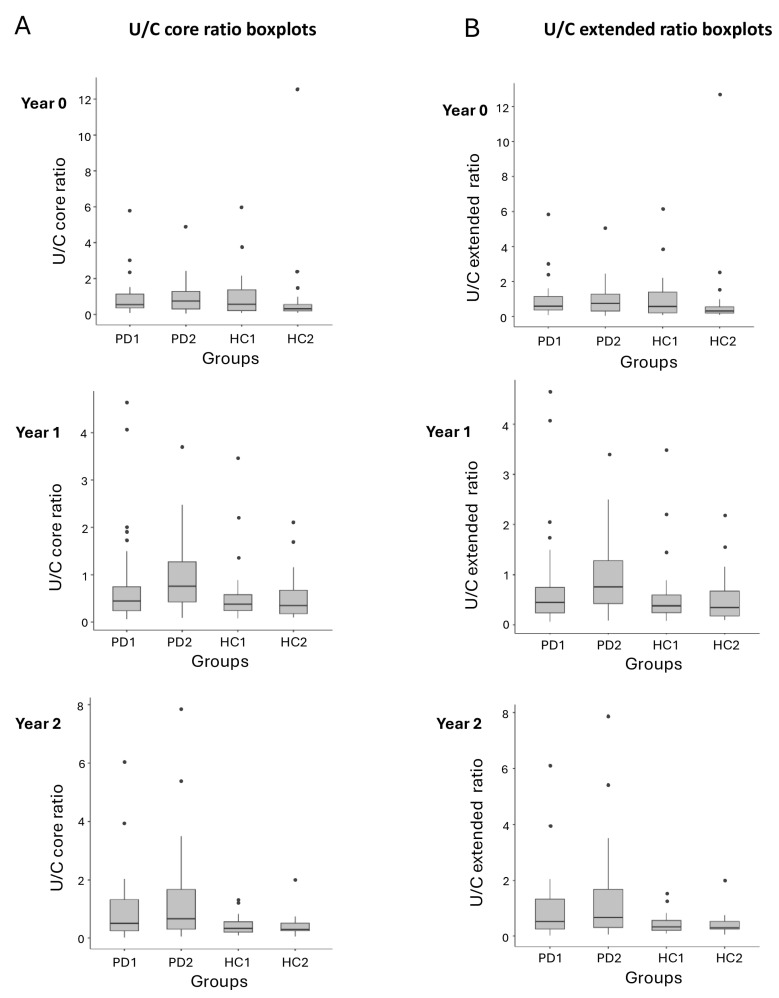
Unconjugated/conjugated bile acid ratio across study groups. Boxplots represent median, interquartile range, and outliers for core (**A**) and extended (**B**) U/C ratio models across three time points (T0–T2). Group differences were assessed using the Kruskal–Wallis test, comparisons between 4 groups were performed using Dunn’s post hoc pairwise comparisons with Bonferroni correction, which did not reveal any statistically significant differences between these individual groups.

**Figure 4:**
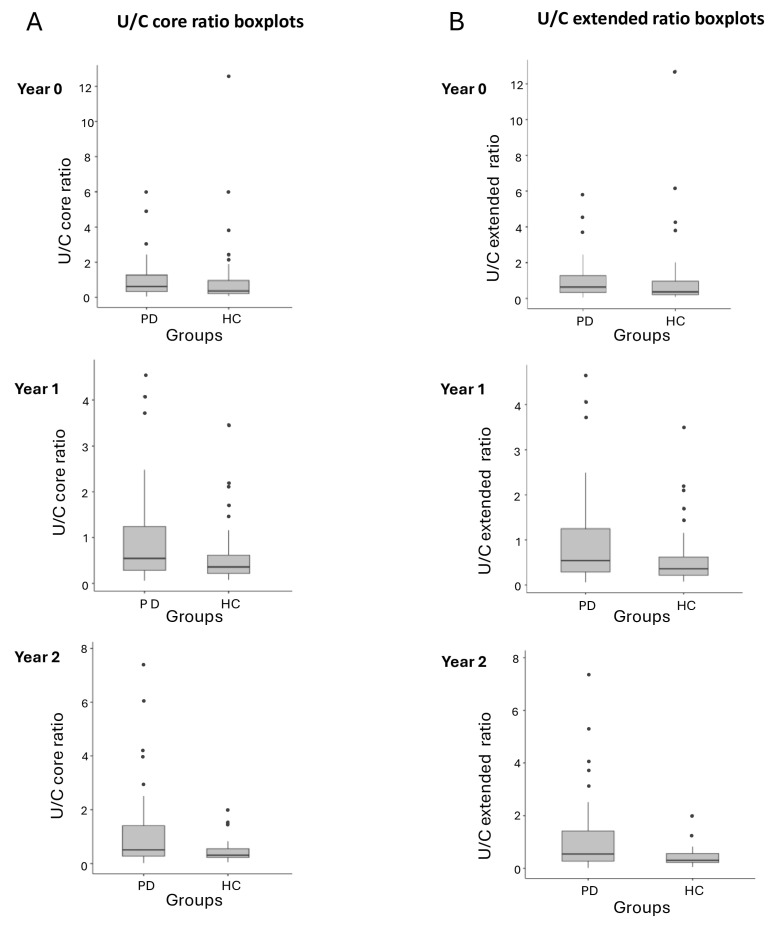
Unconjugated/conjugated bile acid ratio across study groups. Boxplots represent median, interquartile range, and outliers for core (**A**) and extended (**B**) U/C ratio models across three time points (T0–T2). Group differences between pooled PD patients and controls were assessed using the Mann–Whitney U test.

No significant differences in the U/C bile acid ratio were observed between the four groups at any time point using the Kruskal–Wallis test (T0: *p* = 0.153; T1: *p* = 0.093; T2: *p* = 0.070). Consistently, Dunn’s post hoc pairwise comparisons with Bonferroni correction did not reveal any statistically significant differences between individual groups (Figure 3). Given the lack of separation between individual subgroups, further analysis was performed by pooling subjects into patient (PD1 + PD2) and control (HC1 + HC2) groups (Figure 4). This analysis revealed a significant difference in the U/C ratio at T1 (*p* = 0.039) and T2 (*p* = 0.009), while a trend toward significance was observed at T0 (*p* = 0.064).

Inclusion of muricholic BA in the extended ratio model yielded comparable results, with minimal changes in statistical significance (T0: *p* = 0.137; T1: *p* = 0.111; T2: *p* = 0.073). Analysis by pooling subjects into patient (PD1 + PD2) and control (HC1 + HC2) groups also with muricholic acids showed similar results (T0: *p* = 0.054; T1: *p* = 0.049; T2: *p* = 0.010). Overall, while no differences were detected between individual subgroups, a consistent shift in the U/C bile acid ratio was observed between PD patients and healthy controls.

### 3.3. Hydrophobic/Hydrophilic Bile Acids Ratio

**Figure 5:**
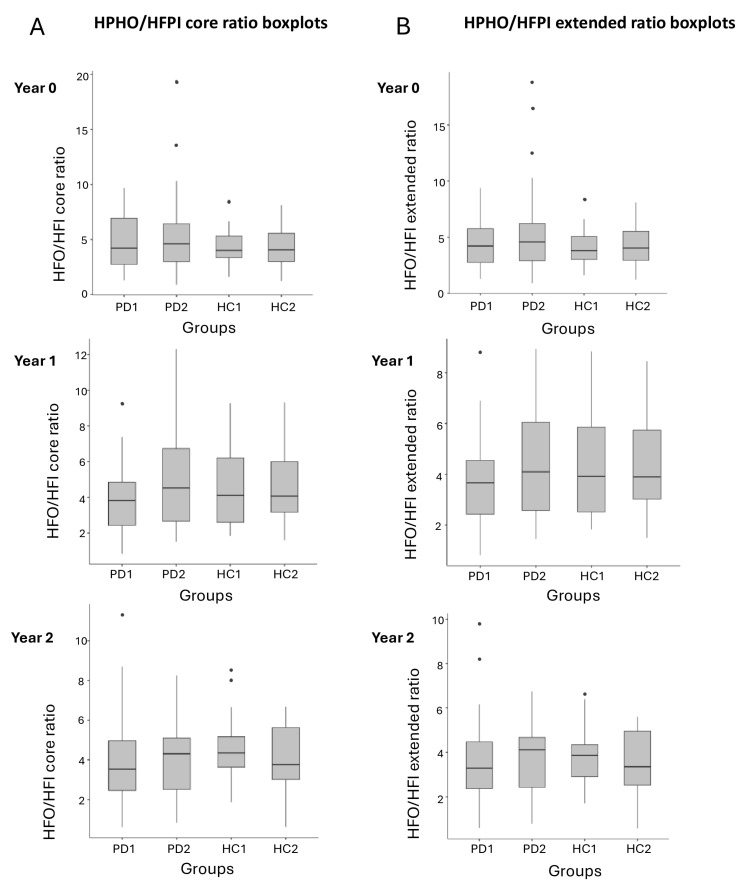
Hydrophobic/hydrophilic bile acid ratio across study groups. Boxplots represent median, interquartile range, and outliers for core (**A**) and extended (**B**) HFO/HFI ratio models across three time points (T0–T2). Group differences were assessed using the Kruskal–Wallis test; comparisons between all 4 groups were performed using Dunn’s post hoc pairwise comparisons with Bonferroni correction, which did not reveal any statistically significant differences between these individual groups.

**Figure 6:**
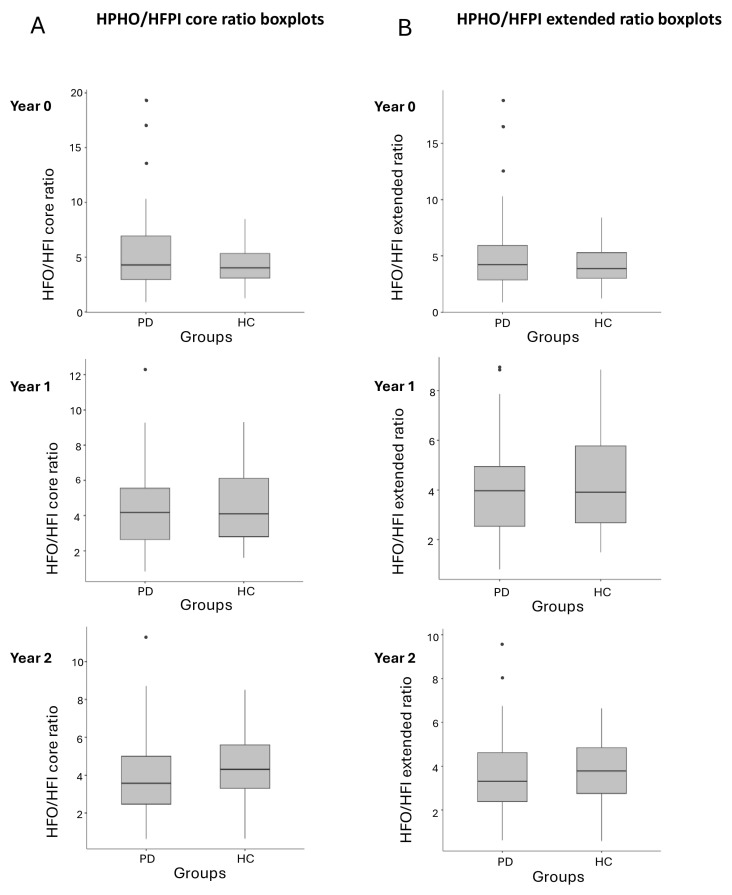
Hydrophobic/hydrophilic bile acid ratio across study groups. Boxplots represent median, interquartile range, and outliers for core (**A**) and extended (**B**) ratio models across three time points (T0–T2). Group differences in pooled comparisons between PD patients and controls were performed using the Mann–Whitney U test.

Analysis of the hydrophobic-to-hydrophilic bile acid ratio did not reveal significant differences between the four groups at any time point using the Kruskal–Wallis test (T0: *p* = 0.654; T1: *p* = 0.475; T2: *p* = 0.459 for the core ratio) (Figure 5). Comparable results were obtained for the extended ratio (T0: *p* = 0.641; T1: *p* = 0.500; T2: *p* = 0.908). Dunn’s post hoc pairwise comparisons with Bonferroni correction did not reveal any statistically significant differences between groups at any time point. Given the lack of separation between individual subgroups, further analysis was performed by pooling subjects into patient (PD1 + PD2) and control (HC1 + HC2) groups (Figure 6), but there is also no significance (T0: *p* = 0.264; T1: *p* = 0.459; T2: *p* = 0.140) for the core ratio and (T0: *p* = 0.279; T1: *p* = 0.518; T2: *p* = 0.598) for the extended ratio.

### 3.4. Associations Between Individual Bile Acids and Their Metabolic Pathways

Associations between individual BA were assessed using Spearman correlation network analysis generated with Metabolite AutoPlotter v2.6. This approach was used to evaluate not only changes at the level of individual metabolites but also alterations in the relationships between BA and the overall organization of their metabolism.

**Figure 7:**
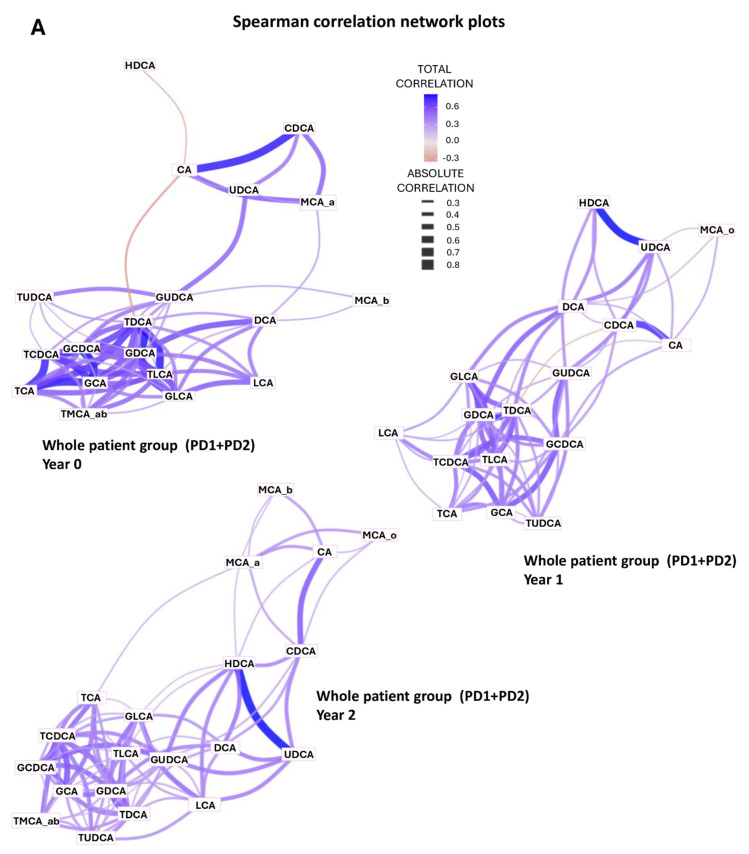

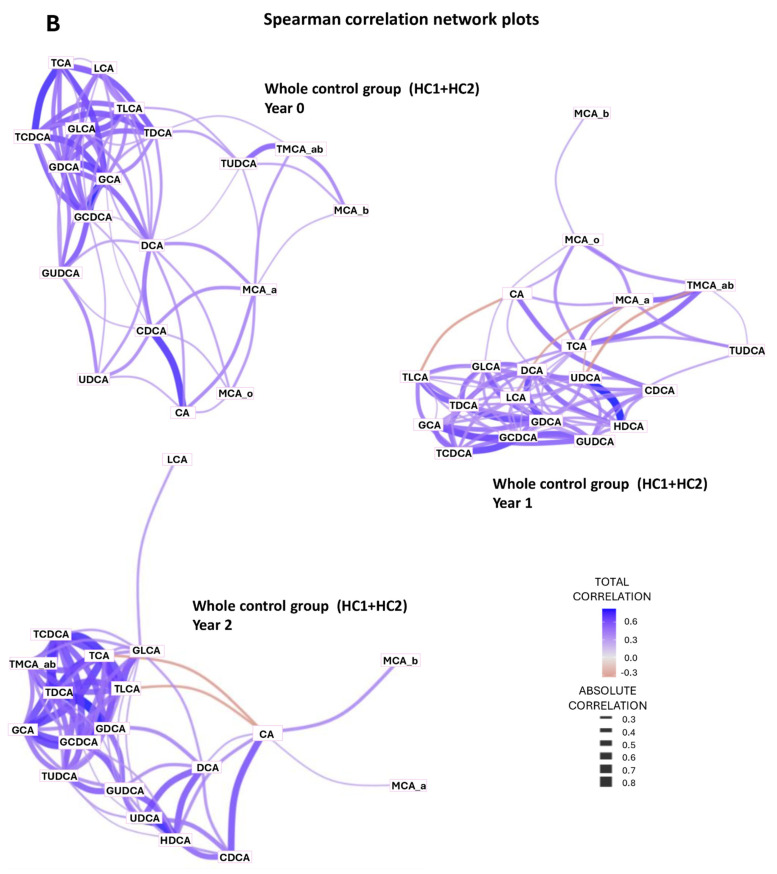
Spearman correlation networks of BA across time (T0–T2) in (**A**) pooled PD (PD1 + PD2) groups and (**B**) control (HC1 + HC2) groups. Nodes represent individual BA, and edges represent pairwise correlations. Edge thickness corresponds to the strength of the correlation, while color indicates direction (blue/purple—positive; red/orange—negative). In both groups, BA form densely interconnected networks dominated by positive correlations, with a central cluster of strongly associated metabolites preserved across time points. Differences between the PD and control groups were limited to minor variations in the strength and distribution of individual correlations.

Correlation networks were constructed for the pooled PD and the pooled HC groups across all three time points. The resulting correlation network plots (Figure 7, and Table 5) illustrate the strength and direction of associations between individual BA within each group.

Spearman correlation analysis identified strong positive associations between several BA pairs in both the pooled PD and the pooled HC groups across individual study years. In both groups, the majority of BA formed a highly connected core network characterized by strong positive correlations, particularly among conjugated and secondary BA. This core structure was preserved across all time points. In the PD group, the strongest correlations in year 0 (T0) were observed for GCA-GCDCA (ρ = 0.830), GDCA-TDCA (ρ = 0.818), and TDCA-TLCA (ρ = 0.813). In year 1 (T1), the top associations were HDCA-UDCA (ρ = 0.926), GDCA-TDCA (ρ = 0.892), and CA-CDCA (ρ = 0.851). In year 2 (T2), the strongest correlations were HDCA-UDCA (ρ = 0.983), GCDCA-TDCA (ρ = 0.774), and GDCA-TDCA (ρ = 0.771).

In the HC group, the strongest correlations in baseline (T0) were observed for GCA-GCDCA (ρ = 0.895), TCA-TCDCA (ρ = 0.853) and CA-CDCA (ρ = 0.834). In year 1 (T1), the top associations were HDCA-UDCA (ρ = 0.879), GCA-GCDCA (ρ = 0.794), and GCDCA-TCDCA (ρ = 0.783), and in year 2 (T2), the strongest correlations were GDCA-TDCA (ρ = 0.850), HDCA-UDCA (ρ = 0.835), and TCA-TCDCA (ρ = 0.807).

Across both groups, recurrent associations were observed for GDCA-TDCA, GCA-GCDCA, and HDCA-UDCA. In the PD group, HDCA-UDCA represented the strongest correlation in T1 and T2, whereas in HC, recurrent strong associations were observed for GCA-GCDCA and TCA-TCDCA. Overall, the correlation structure was largely preserved between PD and HC groups, with only minor differences in the ranking of the strongest BA pairs across study years.

### 3.5. Associations Between Individual Bile Acids and PD Clinical Parameters

Longitudinal analysis using a hierarchical linear mixed model (HLMM) applied to scaled (standardized) values of BA concentrations revealed a non-random and stage-dependent structure of associations with clinical parameters of PD. Given that all variables were standardized prior to modeling the reported estimates represent standardized effect sizes. Specifically, the estimate reflects the expected change (in standard deviation units) in BA concentration associated with a one standard deviation increase in the given clinical parameter, while accounting for repeated measurements and subject-specific variability. The sign of the estimate indicates the direction of the association, with positive values reflecting direct relationships and negative values indicating inverse relationships. The model accounted for repeated measurements across three time points (T0–T2) with subject-specific random effects. A clear asymmetry was observed between disease stages, with the majority of significant associations detected in the PD1 group, whereas PD2 exhibited only sparse and more selective relationships. This pattern suggests that bile acid–clinical coupling is most pronounced in earlier disease phases and becomes progressively attenuated with disease advancement.

**Figure 8:**
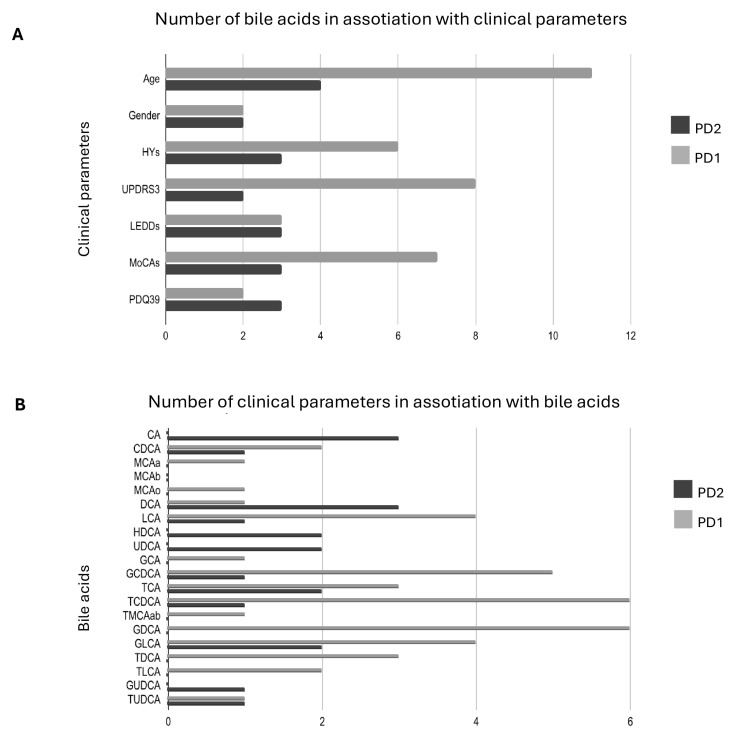
Associations between BA and clinical parameters in Parkinson’s disease. Significant associations were identified using a hierarchical linear mixed model (HLMM) across three time points (T0–T2) with subject-specific random effects (*p* < 0.05). (**A**) Number of BA associated with each clinical parameter. The highest number of associations was observed for age, followed by disease severity (HY, UPDRS3) and cognitive function (MoCA), while gender showed minimal associations. (**B**) Number of clinical parameters associated with individual BA. Conjugated and secondary BA (GCDCA, TCDCA, GDCA, GLCA) accounted for the most associations, whereas primary and muricholic BA showed limited involvement. A higher density of associations was consistently observed in PD1 compared to PD2.

The observed associations were not evenly distributed across all bile acid species but were driven predominantly by conjugated, relatively hydrophobic BA. Glycine- and taurine-conjugated species such as GCDCA, GDCA, and TCDCA exhibited the most consistent and multidimensional relationships across clinical domains (Figure 8). For example, GCDCA in PD1 showed a strong positive association with HY stage (*p* = 0.001; estimate = 0.501) and PDQ39 (*p* < 0.001; estimate = 0.635), alongside a pronounced negative association with UPDRS3 (*p* < 0.001; estimate = −0.891) and a positive association with MoCA (*p* = 0.011; estimate = 0.359). Similarly, GDCA was positively associated with HY stage (*p* = 0.006; estimate = 0.189) and MoCA (*p* < 0.001; estimate = 0.404), while showing negative associations with UPDRS3 (*p* < 0.001; estimate = −0.293) and LEDDs (*p* < 0.001; estimate = −0.278). TCDCA further supported this pattern, being associated with HY stage (*p* = 0.019; estimate = 0.042), UPDRS3 (*p* = 0.018; estimate = −0.045), MoCA (*p* = 0.005; estimate = 0.044), and PDQ39 (*p* = 0.012; estimate = 0.062).

In contrast, unconjugated and more hydrophilic BA displayed fewer and less consistent associations. For instance, DCA showed a modest negative association with UPDRS3 (*p* = 0.038; estimate = −0.121) and age (*p* = 0.047; estimate = −0.014), while LCA, one of the few secondary BA with consistent signals, demonstrated associations with age in both PD1 (*p* < 0.001; estimate = 0.002) and PD2 (*p* = 0.008; estimate = 0.001), suggesting partial preservation of specific metabolic relationships across disease progression.

The directionality of associations revealed a complex and domain-specific pattern. Several conjugated BA exhibited inverse relationships with motor severity (e.g., negative association with UPDRS3) while simultaneously showing positive associations with cognitive performance (MoCA) and quality of life (PDQ39). This multidirectional profile indicates that bile acid-related metabolic signals may differentially track motor and non-motor dimensions of the disease and not reflect a single axis of progression.

In PD2, only a limited subset of associations remained significant. For example, UDCA was negatively associated with UPDRS3 (*p* = 0.011; estimate = −0.079) and MoCA (*p* = 0.032; estimate = −0.043), while TUDCA showed a positive association with LEDDs (*p* = 0.026; estimate = 0.064) and a negative association with MoCA (*p* = 0.001; estimate = −0.017). Additionally, CDCA retained associations with LEDDs (*p* = 0.014; estimate = 0.170), indicating that specific BA remain linked to treatment-related parameters even in later stages.

These findings indicate that relationships between BA and clinical parameters in PD are strongly stage-dependent and preferentially driven by conjugated, hydrophobic BA with broad associations across motor, cognitive, and patient-reported domains. The marked reduction of these associations in PD2 points to a progressive disruption or decoupling of this metabolic network with advancing disease.

## 4. Discussion

This study aimed to characterize longitudinal changes in BA profiles in patients with PD and controls and to explore changes between groups and potential associations with disease progression. Using a combined cross-sectional and longitudinal approach across three time points, we quantified a broad panel of 20 BA and evaluated their temporal dynamics in relation to clinical stratification.

Our results indicate that BA metabolism in PD may involve subtle, distributed alterations, not stable changes in individual metabolites. Although statistically significant longitudinal differences were observed for a limited number of BA, these changes were not consistent across time points and did not follow a clear pattern. Instead, our findings support a gradual remodeling of BA metabolism during disease progression reflected in coordinated shifts across the metabolic network that may not be captured by single-metabolite analyses. This interpretation is consistent with the concept that metabolomic alterations in complex diseases may be more apparent at the level of metabolic patterns, variability, and inter-metabolite relationships than at the level of single markers [31,32,33,34,35,36]. Such an interpretation aligns with growing evidence that PD is associated with systemic metabolic and microbiome-related dysregulation [19,20,37], which is better captured at the pathway level through coordinated changes across metabolite networks than focusing on individual markers [34,35,36].

In the present study, several BA showed directional changes in PD groups, including changes in primary BA and hydrophobicity-related patterns, although these findings were not statistically significant. Therefore, these observations should be interpreted cautiously and considered exploratory. Such patterns may be compatible with altered BA synthesis, enterohepatic circulation, or receptor-mediated feedback regulation involving pathways such as FXR and TGR5 [25,38,39,40]. However, these mechanisms were not directly assessed in the present study. Previous studies have also suggested altered BA metabolism and signaling in PD and other neurodegenerative diseases, supporting the biological plausibility of these observations [41,42].

Muricholic acids were detected in our cohort as low-abundance BA species. These metabolites have been characterized mainly in experimental and microbiome-related contexts and may interact with FXR-related signaling pathways [8,9]. However, their concentrations in human plasma are typically low, and their biological relevance in humans is not completely understood. Although BA signaling through receptors such as FXR and TGR5 has been linked to lipid and glucose metabolism, inflammation and cellular stress responses, receptor activity and downstream signaling were not assessed in the present study [43,44,45]. Thus, any connection between the observed BA profile and FXR/TGR5-mediated mechanisms remains speculative and requires further experimental validation.

At the same time, selected hydrophobic BA species, particularly CDCA-related metabolites, showed relative stability or mild directional decreases in our cohorts [38,46,47]. This observation may be relevant because hydrophobic BA such as LCA and DCA have been associated in experimental studies with cytotoxic effects, including mitochondrial membrane perturbation, loss of mitochondrial membrane potential, and the induction of oxidative stress [22,47]. In contrast, more hydrophilic BA, particularly UDCA and TUDCA, have been described as cytoprotective and anti-apoptotic molecules, with reported effects on mitochondrial stabilization in experimental models [48,49]. UDCA and TUDCA are also being investigated as potential therapeutic candidates in PD, including studies focused on mitochondrial target engagement [50]. Nevertheless, the present data do not provide direct evidence for a compensatory detoxification response or reduced metabolic toxicity. The hydrophobic/hydrophilic BA ratio did not differ significantly between groups. The observed directional patterns should be viewed as hypothesis-generating and biologically plausible, not as mechanistic evidence.

The gut microbiome plays a key role in BA metabolism [5,9]. Secondary BA are formed predominantly through bacterial transformation of primary BA in the intestine, including deconjugation, redox-reactions, and 7α-dehydroxylation, and their high inter-individual variability reflects heterogeneity in microbial composition and functional capacity [6,12]. In the context of PD, this variability is relevant because gut microbiome alterations and BA-related signaling have been increasingly implicated in gut-brain axis dysfunction [14,19,20]. Interactions between BA and the gut microbiome may also influence intestinal barrier function and inflammatory signaling and have been linked to α-synuclein pathology in previous studies [26]. The observed changes were more pronounced in advanced disease stages, suggesting progressive alterations in BA metabolism. However, substantial variability limits their use as stand-alone clinical biomarkers [51,52].

Cluster analysis showed that changes in BA metabolism do not occur uniformly but are organized into several groups with distinct dynamics [50,51]. Some clusters exhibited early changes followed by stabilization, which showed early changes followed by relative stabilization, whereas others showed gradual increases over time, particularly in patients, and may be more sensitive to disease progression [50,51]. This supports the view that different groups of BA reflect distinct regulatory mechanisms, including synthesis, enterohepatic circulation, conjugation, microbial transformation, and detoxification pathways [3,4,6,9,12]. This clustering pattern is consistent with ratio-based analyses, supporting the concept of coordinated remodeling of BA metabolism instead of isolated changes in individual metabolites.

Analysis of the ratio of unconjugated to conjugated BA showed that differences between PD patients and controls were more evident at the level of functional BA ratios, supporting the concept that BA alterations in PD are distributed across the BA metabolic profile rather than confined to individual metabolites [9,12,53]. These results were comparable between two analytical approaches, a “core” model with classical BA and an “extended” model, which additionally included low-abundance BA such as muricholic acids and HDCA. The inclusion of these minor BA did not substantially change the results or their interpretation, indicating that the observed differences were not driven solely by these low-abundance metabolites [3,4,7,8]. The lower unconjugated/conjugated ratio observed in PD is consistent with a relative shift toward increased BA conjugation. Conjugated forms are more soluble because conjugation with glycine or taurine lowers pKa, increases the fraction of ionized species, and generally reduces passive membrane permeability, which may be associated with lower cytotoxicity compared with unconjugated forms [4,23]. BA conjugation and deconjugation are influenced by hepatic metabolism, amino acid availability, and gut microbial activity, linking this ratio to host-microbiome interactions [3,6,12]. In the context of PD, where systemic inflammation and dysregulation of the gut–brain axis have been reported, this shift may reflect altered interactions between host metabolism and the gut microbiome. However, because microbiome composition, hepatic conjugation capacity, and inflammatory mediators were not directly measured in this study, the underlying mechanism remains unclear [19,20].

The ratio of hydrophobic to hydrophilic BA did not show statistically significant differences between groups in either the “core” or “extended” model. However, the present data do not support a firm conclusion regarding altered BA hydrophobicity in PD; some individual BA and clustering patterns showed directional hydrophobicity-related variation, which may be biologically relevant given that BA hydrophobicity influences membrane interactions, cytotoxicity, and mitochondrial stress responses [4,22,23]. Correlation analysis further suggested that differences between PD and control groups may be involved not only in individual BA concentrations but also relationships among BA species. In healthy controls, these relationships appeared relatively stable and were consistent with coordinated physiological regulation of BA metabolism, including coordinated processes of synthesis, transport and enterohepatic circulation [3,4,9].

In PD groups, the overall BA correlation network structure remained largely preserved, but we observed selective variations in the strength and organization of specific metabolic relationships. These changes appeared more evident among primary BA. Because primary BA are influenced by hepatic synthesis and feedback regulation, these patterns may be biologically relevant.

Correlations among conjugated BA were relatively stable across groups, which may indicate that relationships within this BA subgroup were less affected than those involving primary or secondary BA. However, hepatic conjugation capacity was not directly measured and therefore cannot be inferred from correlation patterns alone [3,4,54]. The greatest variability was observed among secondary BA, which showed less consistent correlations across time and groups. Since secondary BA are strongly influenced by microbial metabolism, this finding is compatible with a potential contribution of the gut microbiome-related processes [5,6,9,12,19,20].

Overall, our data demonstrate that while the global architecture of the BA metabolic network remains largely preserved in PD, subtle and localized disruptions occur within specific metabolite interactions. Future longitudinal studies combining BA profiling with gut microbiome analysis and functional metabolic readouts will be needed to clarify the relevance of these findings.

Associations between BA and clinical parameters appeared more pronounced in earlier-stage PD group. In the early-stage group (PD1), significant associations were more numerous and predominantly involved conjugated BA. Because conjugated BA are influenced by enterohepatic circulation and gut microbial activity, these findings may reflect the involvement of multiple regulatory levels [3,5,9]. The non-uniform relationships between BA and clinical parameters suggest that BA metabolism is unlikely to reflect a single linear axis of disease severity but may instead capture different aspects of the clinical phenotype [14,16]. In later stages, a reduction in these associations may indicate a progressive loss of coordination between BA metabolism and the clinical phenotype [51,52].

In PD2, fewer significant associations were observed, which may indicate weaker coupling between BA profiles and clinical parameters at more advanced disease stages, but this interpretation should be considered exploratory, particularly given the variability of BA concentrations and the limited sample size within subgroups. The persistence of selected associations in PD2 suggests that some BA–clinical relationships may remain detectable, but larger longitudinal studies are needed to determine whether these patterns reflect disease-stage-related metabolic changes or cohort-specific variability [33,51,52,53].

Taken together, the findings point to subtle and distributed changes in BA metabolism in PD rather than large alterations in individual metabolites. This indicates that BA profiling may be more informative when interpreted at the level of metabolic patterns and relationships not only isolated BA species. Although the present data do not support the use of BA as standalone biomarkers of PD, they may contribute to a broader metabolic characterization of the disease and provide complementary information on systemic metabolic alterations.

This study has several limitations that should be considered when interpreting the results. First, the relatively high inter-individual variability in BA concentrations, particularly within the patient groups, together with subgroup stratification, reduced the sample sizes and may have limited the detection of subtle but biologically relevant differences.

Second, although the longitudinal design represents a strength of this study, the inclusion of three time points over a three-year follow-up may not fully capture the complex and potentially non-linear dynamics of BA metabolism during the long clinical course of PD (the median survival in idiopathic PD is reported to be approximately 15–20 years from diagnosis [54,55]). Therefore, the observed longitudinal patterns should be interpreted as general trends rather than as precise temporal trajectories.

Third, direct gut microbiome profiling was not performed. Because the gut microbiome plays a central role in BA deconjugation and secondary BA formation, the relative contributions of hepatic regulation, enterohepatic circulation, and microbial metabolism to the observed BA patterns could not be disentangled.

Fourth, information on lifestyle factors, including physical activity, exercise intensity, diet, or sedentary behavior, was not available. Although motor impairment was assessed using HY stage and UPDRS3, these clinical measures cannot substitute for objective or questionnaire-based assessment of physical activity.

Fifth, medication data were available for PD patients, and LEDD was included in the PD-specific longitudinal clinical model. However, detailed information on individual drug doses, treatment duration, and medication changes during follow-up was incomplete. Thus, the potential influence of individual pharmacological interventions on BA profiles could not be fully assessed.

Future studies integrating BA profiling with gut microbiome analysis, detailed medication and lifestyle data, metabolic parameters, and functional or interventional approaches will be needed to clarify the biological relevance of these findings.

Despite these limitations, the longitudinal design and combined analysis of individual BA, functional ratios, and correlation patterns provide a useful framework for further investigation of BA metabolism in PD.

## 5. Conclusions

This study provides longitudinal profiling of plasma bile acids in patients with Parkinson’s disease and age- and sex-matched healthy controls. The results do not show large and consistent changes in individual metabolites but point to subtle alterations in BA composition, functional ratios and relationships between selected BA species. The most consistent group-level finding was observed for the unconjugated to conjugated BA ratio in pooled PD versus control cohorts, whereas the hydrophobic to hydrophilic ratio did not differ significantly between groups. These findings support the relevance of BA profiling as a complementary approach for studying systemic metabolic alterations in PD. The observed patterns are compatible with altered host–microbiome and gut–liver metabolic regulation, but the underlying mechanisms remain to be clarified in future studies integrating BA analysis with integrated multi-omic and clinical datasets.

Based on the present data, BA are unlikely to serve as standalone biomarkers of PD. However, they may provide complementary information on the metabolic phenotype of the disease and help define future research directions focused on gut–liver–brain axis interactions in PD.

## Figures and Tables

**Table 1:** Demographic and clinical characteristics of patients with early- and advanced-stage PD and healthy controls.

Parameter	PD1 Group	PD2 Group	HC1 Group	HC2 Group	*p*-Values
***N* (subjects)**	29	28	28	28	-
**Sex (M%/F%)**	21 (72.4%)/8 (27.6%)	20 (71.4%)/8 (28.6%)	20 (71.4%)/8 (28.6%)	21 (75.0%)/7 (25.0%)	0.989
**Age (years)—mean + SD**	67.1 ± 11.5	69.6 ± 8.4	67.3 ± 10.3	70.2 ± 10.8	0.581
**Clinical Parameters**	**PD1 Group**	**PD2 Group**	**HC1 Group**	**HC2 Group**	***p*-Values**
**HY scale**	1.5 ± 0.4	2.2 ± 0.4	-	-	<0.001
**UPDRS3**	22.2 ± 7.8	46.5 ± 11.3	-	-	<0.001
**LEDD (mg per day)**	487.1 ± 365.7	624.9 ± 555.1	-	-	0.270
**MoCA**	25.9 ± 3.0	25.4 ± 2.1	-	-	0.560
**PDQ39**	26.9 ± 16.0	45.1 ± 22.4	-	-	<0.001

**Table 2:** Classification of BA included in unconjugated and conjugated categories used for U/C ratio calculation. Bile acids marked as “extended” were included only in the extended U/C ratio model.

Unconjugated Bile Acids	Conjugated Bile Acids
Cholic acid (CA)	Glycocholic acid (GCA)
Chenodeoxycholic acid (CDCA)	Glycodeoxycholic acid (GDCA)
Deoxycholic acid (DCA)	Glycolitocholic acid (GLCA)
Lithocholic acid (LCA)	Glycochenodeoxycholic acid (GCDCA)
Ursodeoxycholic acid (UDCA)	Glycoursodeoxycholic acid (GUDCA)
α -muricholic acid (MCAα) (extended)	Taurocholic acid (TCA)
Β-muricholic acid (MCAβ) (extended)	Taurodeoxycholic acid (TDCA)
ω-muricholic acid (MCAω) (extended)	Taurolithocholic acid (TLCA)
Hyodeoxycholic acid (HDCA) (extended)	Taurochenodeoxycholic acid (TCDCA)
	Tauroursodeoxycholic acid (TUDCA)
	Tauromuricholic acids (TMCAα+β) (extended)

**Table 3:** Classification of BA included in hydrophobic and hydrophilic categories used for HFO/HFI ratio calculation. BA marked as “extended” were included only in the extended HFO/HFI ratio model.

Hydrophobic Bile Acids	Hydrophilic Bile Acids
Chenodeoxycholic acid (CDCA)	Glycocholic acid (GCA)
Glycochenodeoxycholic acid (GCDCA)	Taurocholic acid (TCA)
Deoxycholic acid (DCA)	Cholic acid (CA)
Lithocholic acid (LCA)	Ursodeoxycholic acid (UDCA)
Taurochenodeoxycholic acid (TCDCA)	Glycoursodeoxycholic acid (GUDCA)
Glycodeoxycholic acid (GDCA)	Tauroursodeoxycholic acid (TUDCA)
Glycolithocholic acid (GLCA)	α -muricholic acid (MCAα) (extended)
Taurolithocholic acid (TLCA)	β-muricholic acid (MCAβ) (extended)
Taurodeoxycholic acid (TDCA)	ω-muricholic acid (MCAω) (extended)
	Tauromuricholic acids (TMCAα+β) (extended)
	Hyodeoxycholic acid (HDCA) (extended)

**Table 4:** Selected BA showing changes in mean concentrations (µmol/L) between time points (T0–T1, T0–T2, and T1–T2) in patient (PD1, PD2) and control (HC1, HC2) groups. Delta (Δ) values represent differences in mean concentrations between the respective time points within each group. Corresponding *p*-values indicate the statistical significance (if *p* < 0.05) of these temporal changes.

Bile Acids	Groups	∆T0–T1 in µmol/L	∆T0–T2 in µmol/L	∆T1–T2 in µmol/L	*p* Value ∆T0–T1	*p* Value ∆T0–T2	*p* Value ∆T1–T2
CA	HC1	0	−0.08	−0.08	0.979	0.716	0.736
	HC2	0.04	0.05	0.01	0.556	0.413	0.818
	PD1	−0.03	0.1	0.13	0.803	0.399	0.275
	PD2	−0.06	0.13	0.19	0.588	0.295	0.113
CDCA	HC1	−0.05	−0.16	−0.11	0.811	0.474	0.634
	HC2	0.05	0	−0.05	0.708	0.978	0.729
	PD1	0.2	0.02	−0.18	0.296	0.927	0.339
	PD2	0.38	0.14	−0.24	0.013	0.362	0.118
LCA	HC1	0.031	0.028	−0.003	0.001	0.002	0.739
	HC2	0.04	0.02	−0.02	<0.001	0.022	0.028
	PD1	0.05	0.03	−0.02	<0.001	0.007	0.16
	PD2	0.06	0.02	−0.04	<0.001	0.065	<0.001
UDCA	HC1	−0.03	0.05	0.08	0.663	0.568	0.317
	HC2	0.05	0.02	−0.03	0.595	0.798	0.782
	PD1	0.12	0.01	−0.11	0.146	0.872	0.204
	PD2	0.17	0.08	−0.09	0.007	0.208	0.153

**Table 5:** Spearman correlation analysis identified the TOP 3 strongest positive associations between several BA pairs in both the pooled PD and pooled HC groups across individual study years.

Group	Year	Correlation Relationship	ρ Coeff.	Group	Year	Correlation Relationship	ρ Coeff.
PD	0	GCA-GCDCA	0.830	HC	0	GCA-GCDCA	0.895
		GDCA-TDCA	0.818			TCA-TCDCA	0.853
		TDCA-TLCA	0.813			CA-CDCA	0.834
PD	1	HDCA-UDCA	0.926	HC	1	HDCA-UDCA	0.879
		GDCA-TDCA	0.892			GCA-GCDCA	0.794
		CA-CDCA	0.851			GCDCA-TCDCA	0.783
PD	2	HDCA-UDCA	0.983	HC	2	GDCA-TDCA	0.850
		GCDCA-TCDCA	0.774			HDCA-UDCA	0.835
		GDCA-TDCA	0.771			TCA-TCDCA	0.807

## Data Availability

All data are available from the corresponding author upon reasonable request.

## Abbreviations

The following abbreviations are used in this manuscript:

BA	bile acids
BIC	Bayesian information criteria
CA	cholic acid
cAMP	cyclic adenosine monophosphate
CDCA	chenodeoxycholic acid
DCA	deoxycholic acid
EDTA	ethylenediaminetetraacetic acid
FXR	farnesoid X receptor
GCA	glycocholic acid
GCDCA	glycochenodeoxycholic acid
GDCA	glycodeoxycholic acid
GLCA	glycolithocholic acid
GPCR	membrane G-protein-coupled receptor
GUDCA	glycoursodeoxycholic acid
HC	healthy control
HDCA	hyodeoxycholic acid
HLMM	hierarchical linear mixed model
HY	Hoehn–Yahr scale
LCA	lithocholic acid
LEDD	Levodopa equivalent daily dose
MCAα	α-muricholic acid
MCAβ	β-muricholic acid
MCAω	ω-muricholic acid
MoCA	Montreal cognitive assessment
PD	Parkinson’s disease
PDQ39	Parkinson’s disease questionnaire 39
QC	quality control
SD	standard deviation
TCA	taurocholic acid
TCDCA	taurochenodeoxycholic acid
TDCA	taurodeoxycholic acid
TGR5	Takeda G protein-coupled receptor 5
TLCA	taurolithocholic acid
TMCAα+β	tauromuricholic acids α+β
TUDCA	tauroursodeoxycholic acid
UDCA	ursodeoxycholic acid
UPDRS3	Unified Parkinson’s disease rating scale part 3 (Motor examination)
